# Correlation between Blood Monocytes and CSF Tau in Alzheimer’s Disease: The Effect of Gender and Cognitive Decline

**DOI:** 10.3390/neurosci4040026

**Published:** 2023-12-12

**Authors:** Carlotta Ginevra Valentina Cimiotti, Paolo Paganetti, Stefania Rossi, Emiliano Soldini, Leonardo Sacco

**Affiliations:** 1 Neuropsychological and Speech Therapy Unit, Neurocenter of Southern Switzerland, Ente Ospedaliero Cantonale, CH-6900 Lugano, Switzerland; 2 Faculty of Biomedical Sciences, Università della Svizzera Italiana, CH-6900 Lugano, Switzerland; 3 Laboratory for Aging Disorders, LRT, Ente Ospedaliero Cantonale, CH-6500 Bellinzona, Switzerland; 4 Competence Centre for Healthcare Practices and Policies, Department of Business Economics, Health, and Social Care (DEASS), University of Applied Sciences and Arts of Southern Switzerland, CH-6928 Manno, Switzerland; emiliano.soldini@supsi.ch

**Keywords:** patients, monocytes, tau, cerebrospinal fluid, MoCA, gender difference, cognitive decline, Alzheimer’s disease pathogenesis, diagnostics

## Abstract

Neuroinflammation is one of the main mechanisms contributing to the pathogenesis of Alzheimer’s disease (AD), although its key role and the immune cells involved have not yet been identified. Blood monocytes appear to play a role in the clearance of AD-related amyloid-β (Aβ) and tau protein. This retrospective study evaluated a possible correlation between blood monocytes; the concentrations of Aβ, total tau (t-Tau), and phosphorylated tau (p-Tau) in the cerebrospinal fluid (CSF); and cognitive decline assessed according to the Montreal Cognitive Assessment (MoCA). We collected data from 33 patients with AD or mild cognitive impairment (MCI) due to AD (15 men and 18 women) and found, along with a significant reduction in the concentration of blood monocytes in women (*p*-value = 0.083),significant correlations between the number of blood monocytes and the concentration of t-Tau in CSF (*p*-value = 0.045) and between blood monocytes and MoCA score (*p*-value = 0.037). These results confirm the role of blood monocytes in the pathogenesis of AD, provide further evidence of a gender difference in the neuroinflammatory process underlying AD, and show that blood monocyte count may reflect the cognitive impairment of AD patients.

## 1. Introduction

Alzheimer’s disease (AD) is one of the major health burdens in our society and the main cause of dementia in people older than 65 years, affecting 60–70% of all dementia cases [1,2,3,4]. It is estimated that around 50–55 million people are affected by dementia, corresponding to 0.7–0.75% of the population worldwide [2,5]. In the last thirty years, the prevalence of dementia has more than doubled, and the World Health Organization (WHO) estimates that new cases increase by 10 million each year and will continue to rise in line with the ageing of the population [1,6]. AD is characterized by progressive disability with increasing morbidity, resulting in hurtful consequences for patients and their caregivers as well as mortality. Consequently, the absence of an effective therapy makes this disease a major issue for today’s healthcare system and a considerable contributor to healthcare costs [2,4,5].

AD is a neurodegenerative disease whose pathophysiological process is still under investigation. However, some hypotheses have acquired greater importance and have therefore been extensively studied. Among these, the amyloid cascade has been considered a pathogenic driver of AD [7,8,9]. This hypothesis postulates that AD may result from the accumulation of Aβ protein in the extracellular space in the form of senile plaques [10] and the formation of intracellular neurofibrillary tangles (NFTs) made of fibrillar, hyperphosphorylated tau protein [11]. The latter mechanism may be triggered by pathogenic forms of Aβ [12,13,14,15] causing a loss of communication between neurons as well as the failure of signal processing, culminating in the death of the neurons and the typical clinical scenario of cognitive impairment and changes in personality [7,16,17,18,19,20].

Whilst the amyloid cascade represents the most studied pathophysiological hypothesis for AD, new pathogenetic models are gaining relevance. Among them, neuroinflammation appears to contribute to the pathogenesis of AD by involving various central nervous system (CNS) cell types, ultimately fueling a vicious circle accelerating the neurodegenerative process via mechanisms that are not fully understood [21,22]. Indeed, it is proposed that rather than being secondary to the amyloid cascade, neuroinflammation may contribute at least as much as Aβ and tau to AD pathogenesis [21,22,23,24]. Furthermore, analyses of the first clinical stages of AD and the presence of an inflammatory process (e.g., in cerebrospinal fluid) have shown immune system to play a much earlier role in the pathogenesis of AD, indicating that neuroinflammation may also steer degeneration, independent of plaque deposition [22,24]. A study by Krstic et al. even showed that neuroinflammation may precede or cause senile plaques and NFT accumulation [25].

Unlike conventional neuroinflammatory diseases, such as multiple sclerosis (MS) and encephalitis, in which mainly adaptive immune system cells (T- and B-lymphocytes) are involved [26,27], the inflammatory process of AD primarily engages immune cells residing in the CNS, i.e., the innate immune system of the brain, represented by microglia, astrocytes, and perivascular macrophages [22,28,29]. The recruiting of other immune cells derived from the periphery into the CNS, including monocytes and dendritic cells, is under investigation. It has been suggested that Aβ accumulation could be attributed to failed clearance by macrophages and peripheral monocytes. Hence, unhealthy and dysfunctional bone-marrow-derived monocytes may play a major role in AD pathology [22,24,30,31,32,33,34,35].

Whilst preclinical studies conducted on animal models have highlighted the role of the microglia and the monocyte in the pathogenesis of AD [36,37], few clinical studies have addressed the value of blood monocyte count as a marker of neurodegeneration. A recent prospective study by Sun et al. comprising 127 AD patients and 100 controls showed a significant reduction in blood monocyte count in AD patients compared to controls (*p* < 0.001), which inversely correlated with CSF t-Tau (*p* = 0.019 for AD), p-Tau (*p* = 0.001 for AD), t-Tau/Aβ42, and p-Tau/Aβ42 in 46 AD patients (Spearman’s correlation was used for the statistical analyses and the threshold for significance was set at 5%). These correlations were not found in their control group, and no correlation was found between blood monocytes and Aβ42 (*p* = 0.275) [38]. These data provide evidence of an association between increased blood monocytes and reduced CSF tau, perhaps due to a monocyte-driven clearance function against brain tau. The objective of our study was to confirm these data by performing a retrospective study on a cohort of 33 patients diagnosed with AD or mild cognitive impairment (MCI) due to AD. In addition, we investigated whether blood monocyte count correlated with gender or with the severity of cognitive impairment.

## 2. Materials and Methods

### 2.1. Subjects

A group of N = 33 patients fulfilling the clinical consensus criteria for AD [39] or MCI due to AD [40] were consecutively recruited between 2019 and 2021 from our memory clinic (the Neurocenter of Southern Switzerland) and evaluated by a team of experienced behavioral neurologists and neuropsychologists. Informed consent was obtained from all subjects involved in the study. The sample included 15 men and 18 women, with a median age of 70 years and a median of 13 years of education (see Table 1). Men and women did not differ significantly in terms of age (*p*-value = 0.993) or number of years of education (*p*-value = 0.293). Clinical severity was scored for N = 29 patients, whereby N = 21 underwent a Montreal Cognitive Assessment (MoCA) [41] and N = 8 underwent a mini mental state examination (MMSE) [42]. In order to standardize the cognitive assessment data and enable correlation analyses to be carried out, we used a validated MMSE–MoCA conversion table [43]. The MoCA includes aspects of memory recall, visuospatial ability, executive functions, orientation, language, attention, concentration, and working memory. This comprehensive measure of cognitive functions displays an improved sensitivity at an early stage of the disease, such as in MCI due to AD [41,44,45].

**Figure 1:**
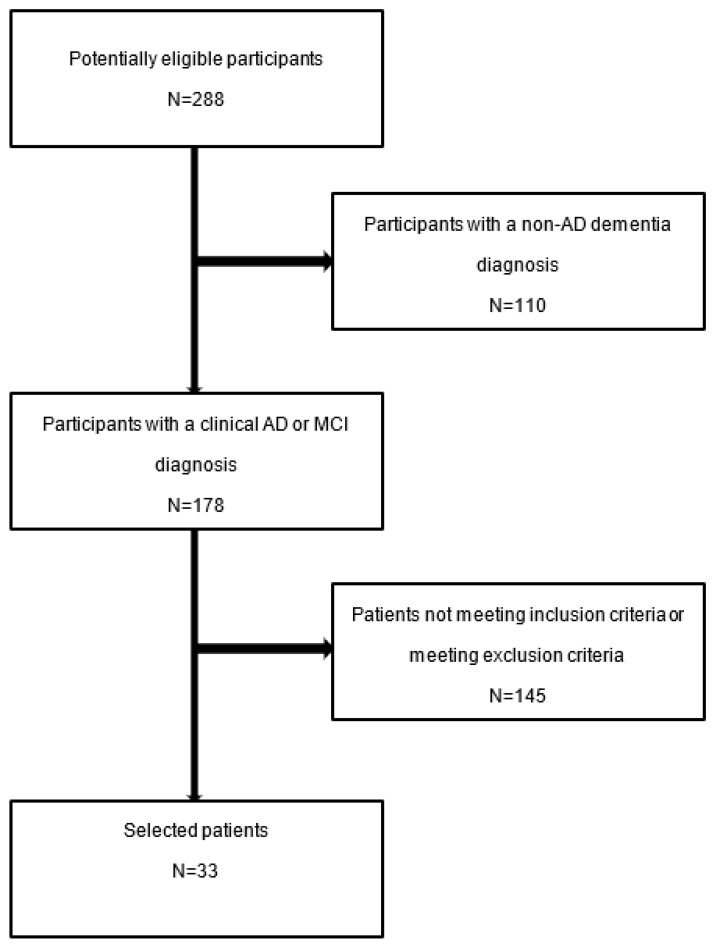
Patient selection flowchart.

According to the currently in-use NIA-AA criteria, revised in 2011 by McKhann et al. [46] and Albert et al. [47], the AD pathophysiological process for the N = 33 patients with available blood monocytic counts (measured using an automatic blood analyzer) included in this study was confirmed in N = 30 using pathological CSF markers (Aβ42 < 780 ng/L, t-Tau > 290 ng/L, p-Tau > 60 ng/L). CSF was collected via lumbar puncture and analyzed by enzyme-linked immunosorbent assay (ELISA by Fujirebio) in two different laboratories; one of them also provided the Aβ40/42 ratio. The three patients with missing CSF data presented typical AD imaging either by amyloid PET, FDG PET, or MRI, again according to the NIA-AA criteria [46,47]. Most patients underwent more than one investigation to confirm the diagnosis. Indeed, MRI was performed on 32 patients, amyloid PET on 5 patients, and FDG PET on 8 patients. Subjects with neoplastic diseases causing myelosuppression or taking myelotoxic drugs were excluded from the study. A flowchart of the patients included is depicted in Figure 1.

We retrospectively identified 288 patients with a clinical diagnosis of dementia investigated at our memory clinic between 2019 and 2021. Of these, 178 had received a clinical diagnosis of AD or MCI. We were compelled to exclude most of them either because they lacked invasive investigations, which are usually not performed for MCI, or because the patients were above 75 years of age. Patients were then excluded if they had: other types of dementia or neurological disorders affecting cognitive function, major psychiatric disorders, a history of severe head trauma or chronic alcohol abuse, severe systemic disorders including immunological ones, or no analysis of the blood monocytes count. The small number of patients selected and the absence of a control group are due to the small size of our memory clinic, the retrospective nature of the study, and the requirement of invasive examination for the CSF proteins.

### 2.2. Statistical Analyses

Continuous variables were described using medians and interquartile ranges, while the only categorical variable (gender) was presented using absolute frequencies. The relations between blood monocytic count and the other continuous variables were assessed through Spearman correlation coefficients, while the relation with gender was evaluated using the Mann–Whitney test. The relations between gender and the CSF AD biomarker levels were also assessed using the Mann–Whitney test. The threshold for statistical significance was set at the 10% to mitigate the high risk of type II errors associated with limited sample size. We performed all statistical analyses with Stata/IC 16.0 (StataCorp, 4905 Lakeway Drive, College Station, TX, USA).

## 3. Results

### 3.1. Description of the Data

Descriptive statistics of the variables considered are reported in Table 1. The median levels of t-Tau and p-Tau were 502 and 109 ng/L, respectively, while the median Aβ42 level equaled 495.5 ng/L. The median Aβ40/42 ratio was 0.07, the median MoCA score was 19, and the median CDR score was 1. The median blood monocyte count was 0.4*10^9^ per liter.

### 3.2. Bivariate Analysis

**Figure 2:**
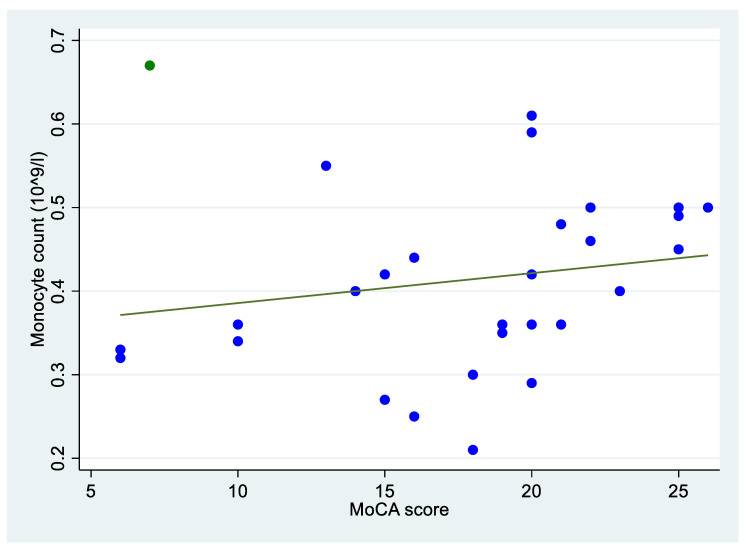
Scatter plot with regression line showing the direct correlation between MoCA score and blood monocyte count (10^9^/L). The green point is considered an outlier.

Table 2 reports the assessment of the relations between blood monocyte count and the other variables considered. The numerical values reported highlight three statistically significant relations. The blood monocyte level was significantly higher among men, with a median of 0.44 × 10^9^ per liter, compared to women, with a median of 0.36 × 10^9^ per liter (*p*-value = 0.083). Moreover, blood monocyte count was inversely correlated with t-Tau level (*p*-value = 0.045) and directly correlated with MoCA score (MoCA *p*-value = 0.037) (Figure 2).

No statistically significant relations between gender and the CSF AD biomarker levels were found (output omitted for brevity).

## 4. Discussion

This study found that CSF t-Tau has an inverse correlation with blood monocyte count. This observation validates an earlier report by Sun et al., who showed a significant negative correlation between CSF t-Tau and blood monocyte level (*p* = 0.019 for AD, *p* = 0.041 for MCI) and between the latter and CSF p-Tau (*p* = 0.001 for AD, *p* = 0.057 for MCI) in AD and MCI patients [38]. The smaller cohort size and the retrospective design of our study emphasizes the robustness of the previous findings. Furthermore, with our analysis of clinical severity and gender as additional variables, we obtained evidence of the impact of blood monocyte count on disease course as well as evidence of lower blood monocyte counts in women affected by AD or MCI due to AD when compared to men.

Neuroinflammation plays a key role in the development of AD, and evidence exists to show that it may contribute to the pathogenesis of AD at least as much as the deposition of Aβ and tau aggregates [21,22,23,24]. Extrinsic factors promoting neuroinflammation (systemic inflammation, obesity, traumatic brain injury) have been reported to increase the risk of developing AD [22,24]. In addition, increased concentration of inflammatory mediators such as interleukin-1 and tumor necrosis factor have been observed in the brains of AD patients and of mice models [37,48,49].

Genome-wide association studies (GWAS) have uncovered a link between a higher risk of AD and variations in the genes encoding for triggering receptor expressed on myeloid cells 2 (TREM2) [50,51] or the myeloid cell surface antigen CD33 [52,53,54]. Notably, TREM2 and CD33 modulate the ability of monocytes to clear Aβ [37,50,51,52,54,55]. Furthermore, neurodegeneration in mice has been shown to be mitigated by, for example, peripheral exposure to myelin-derived antigens, causing the recruitment of monocytes to the brain [37,56,57], which may assist resident immune cells in counteracting protein accumulation [22,24,30,31,32,33,34,35]. Blood-derived monocytes may even substitute impaired resident microglia in the disease [32,58], as shown by the peripheral infusion of activated monocytes [59,60].

Much evidence has been produced on the interaction between microglia and monocytes and the accumulation of Aβ, as these have a possible role in clearing brain Aβ protein, which may also alleviate downstream AD pathologies, including those linked to aberrant tau metabolism. Whilst their action on NFTs remains to be elucidated, the clearance of tau protein by monocytes has been reported [22,61,62,63]. Chemokines are important mediators of immune cell migration to the site of inflammation, including monocyte recruitment to the CNS. In mice, C-C chemokine motif receptor 2 (CCR2) deficiency has been shown to reduce monocyte infiltration, which is linked to AD-like pathology [31,62,64], and increase tau pathology [65]. Paradoxically, microglial C-X3-C motif chemokine receptor 1 (CX3CR1) disruption has been shown to exacerbate tau deposition whilst enhancing the phagocytic ability of microglial against Aβ deposition [22,62,66,67,68,69,70,71]. However, a study by Wang et al. showed that tau removal from the blood by peripheral monocytes may also contribute to slowing down brain accumulation of phosphorylated tau and neurodegenerative damage [72], highlighting once more the importance of peripheral monocytes in AD pathology. Although lacking direct clinical evidence, empowering blood-derived cells to accelerate tau clearance may represent a potential AD treatment [37], a strategy jeopardized by the role of neuroinflammation in fostering neurodegeneration [24,73,74,75], which is perhaps linked to disease progression [62].

The fact that blood monocyte count correlates both with CSF tau concentration and MoCA score supports the hypothesis according to which high CSF tau predicts a rapid cognitive decline [76,77] and is correlated with the intensity of the disease [78,79,80]. However, conflicting results have been produced in this regard, and the role of CSF tau in explaining the link between blood monocytes and cognitive impairment remains only hypothetical.

Our study also proposes gender as another factor influencing blood monocyte count in AD. Remarkably, lower blood monocyte levels were found in women, who are known to suffer a faster cognitive decline in MCI or AD [81]. Although the impact of gender on Aβ and tau burden is unclear [81], a longitudinal study in the Alzheimer’s Disease Neuroimaging Initiative (ADNI) cohort revealed that women with positive CSF biomarkers suffered from faster neurodegeneration and cognitive deterioration than men [82]. Sparce evidence exists for possible mechanisms generating a gender difference in the neuroinflammatory process [83], such as the possible role of microglial miRNAs and their consequences on microglial transcriptome and tau pathogenesis [84]. Interestingly, an AD-linked TREM2 variant leads to a more rapid microglial reaction and a more important memory impairment in female mice compared to male mice. Furthermore, the same TREM2 variant causes increased inflammation only in female mice [83,85], suggesting that inflammation may play a role in the gender differences. [83]. Systematic studies in this field are necessary to better define these aspects, which are critical for developing and implementing precision medicine in AD. Importantly, our findings imply that gender should be considered as a critical modifier when designing predictive models for AD as well as when planning clinical trials. However, gender difference in peripheral monocyte counts may represent an AD-independent characteristic [86].

We also report an inverse correlation between blood monocytes and the severity of the clinical presentation when assessed by MoCA score [41]. To our knowledge, this result is the first to be reported in a population of patients with biologically confirmed AD. The presence of lower monocyte counts in patients with a severe cognitive impairment could be explained by the role of inflammation in the different stages of the disease, with it making a significant contribution when the neurodegeneration progresses [37]. Lower levels of monocytes, and thus the failure of monocyte activity in AD pathology, would be associated with a greater cognitive impairment and consequently with a lower score MoCA score.

The most significant limitation of this study is that the correlation between blood monocyte count and CSF tau does not address the causality of the process and the role, in vivo, of the monocyte. Also, lower monocyte counts in blood correlated with higher CSF tau, but this association was not found for CSF Aβ (however, similar results were found by Sun et al. [38]). Another limitation is the use of a retrospective study design without a control group. These two limitations mean that we did not have biological data without preanalytical and analytical standardization and could only make a comparison with the normal laboratory reference parameters. Indeed, lumbar punctures were not performed at the same time of the day for all the patients, and the CSF analysis was performed at two different laboratories, though these used the same technique and unit of measurement. Furthermore, as mentioned above, some patients only had an MMSE result, which was converted into an MoCA score. Despite these limitations, our results provide further in vivo evidence of the possible implication of the immune system in AD. Changes in immune cells, the interaction between immune cell dysfunctions and AD pathology, and the gene susceptibility of immune cells all relate to AD [87]. We are therefore encouraged to continue our studies, which have the potential to improve the diagnosis of Alzheimer’s disease with a cell-based marker perhaps involved in the pathogenic process.

## 5. Future Perspectives

Our explorative study represents a preliminary step supporting the need for further research focused on inflammatory cells as a potential key player in AD clinical progression. Further research to substantiate our findings are needed, preferably in a prospective setting with a control group and a larger sample size. The link between blood monocytes and tau proteins should be investigated to question the possibility of a direct causal relationship. Relevant CSF mediators of immune cell migration into the CNS should also be analyzed in correlation with conventional CSF protein markers for AD. The impact of gender and perhaps of aging on blood monocyte counts should be confirmed and compared with a control group.

## Figures and Tables

**Table 1:** Descriptive statistics.

Variables	Median (Mean)	Interquartile Range
**Sociodemographics**		
Gender (N = 33)		
*Men* (*count*)	15	-
*Women* (*count*)	18	-
Age (N = 33)	70.00 (70.24)	13.50
Years of education (N = 33)	13.00 (12.30)	4.00
**CSF AD biomarkers**		
t-Tau [ng/L] (N = 30)	502.50	358.50
p-Tau [ng/L] (N = 29)	109.00	69.50
Aβ42 [ng/L] (N = 30)	495.50	245.00
Aβ40/42 ratio (N = 22)	0.07	0.03
MoCA score (N = 29)	19.00 (17.66)	7.00
CDR (N = 33)	1.00 (1.27)	1.50
**Blood monocyte count [10^9^/L]** **(N = 33)**	0.40	0.17

CSF, cerebrospinal fluid; t-Tau, total tau; p-Tau, phosphorylated tau; Aβ, amyloid-β; MoCA, Montreal Cognitive Assessment.

**Table 2:** Relations between blood monocyte count and the other variables.

Variables	Blood Monocyte Count [10^9^/L]		
	Spearman Correlation Coefficients	Mann–Whitney Test	
		Median (IQR)	Test Result
**Sociodemographics**			
Gender (N = 33)			
*Men*	-	0.44 (0.14)	z = −1.739 *
*Women*	-	0.36 (0.19)	
Age (N = 33)	0.237	-	
**CSF AD biomarkers**			
t-Tau [ng/L] (N = 30)	−0.368 **	-	
p-Tau [ng/L] (N = 29)	−0.277	-	
Aβ42 [ng/L] (N = 30)	−0.058	-	
Aβ40/42 ratio (N = 22)	0.091	-	
MoCA score (N = 29)	0.390 **		

** *p* < 0.05, * *p* < 0.1. CSF, cerebrospinal fluid; t-Tau, total tau; p-Tau, phosphorylated tau; Aβ, amyloid-β; MoCA, Montreal Cognitive Assessment.

## Data Availability

The raw data supporting the conclusions of this article will be made available by the authors, without undue reservation.

## Abbreviations

AD	Alzheimer’s disease
Aβ	Amyloid-β protein
Aβ42	Amyloid-β with 42 residues
CCR2	C-C motif chemokine receptor 2
CDR score	Clinical Dementia Rating score
CNS	Central nervous system
CSF	Cerebrospinal fluid
CX3CR1	C-X3-C motif chemokine receptor 1
FDG	[18F] Fluorodeoxyglucose
GWAS	Genome-wide association studies
MCI	Mild cognitive impairment
MMSE	Mini mental state examination
MoCA	Montreal Cognitive Assessment
MRI	Magnetic resonance imaging
MS	Multiple sclerosis
NFTs	Neurofibrillary tangles
NIA-AA	National Institute on Aging and Alzheimer’s Association
p-Tau	Phosphorylated tau
PET	Positron emission tomography
t-Tau	Total tau
TREM2	Triggering receptor expressed on myeloid cells 2
WHO	World Health Organization
